# 

**Published:** 1902-02

**Authors:** 


### CONTENTS.

ORIGINAL ARTICLES:
The Comparative Virulence of the Tubercle Bacillus from Human and Bovine Sources. By MAZYCK P. RAVENEL, M.D. . . . 65
Texas Fever in Native South Carolina Cattle. By G. E. NESOM, D.V.M. . . . 81
The Attitude of the Farmer Toward the Tuberculin Test. By CARL W. GAY, D.V.M. . . . 90
The X-Ray as an Aid in the Diagnosis of Tuberculosis in Cattle. By J. V. LADDEY, D.V.S. 97
Progress in Veterinary Medicine in Its Relation to Public Health. By WILLIAM HERBERT LOWE, D.V.S. . . . 104
ABSTRACTS FROM FOREIGN JOURNALS . . . 109
REPORTS OF CASES:
Urethral Calculi. By J. HUGO REED, V.S. . . . 115
Antitoxin in Distemper. By H. A. STEVENSON, M.D. . . . 116
Creolin (Pearson) in Arthritic Synovitis. By GEORGE MASGANY, V.S. . . . 116
Traumatic Tetanus . . . 117
EDITORIALS:
The Attitude of the Dominion Shorthorn Breeders Toward the Tuberculin Test. . . . 118
What is the Proper Field of the American Veterinary Medical Association? . . . 120
Minneapolis Wins . . . 121
At It Again . . . 122
NECROLOGY . . . 122
COMMUNICATION—SOCIETY PROCEEDINGS—NEW PUBLICATIONS—PERSONALS . 124-132

